# Compatibility between agendas for improving human development and wildlife conservation outside protected areas: Insights from 20 years of data

**DOI:** 10.1002/pan3.10041

**Published:** 2019-08-01

**Authors:** Judith M. Ament, Ben Collen, Chris Carbone, Georgina M. Mace, Robin Freeman

**Affiliations:** ^1^ Institute of Zoology Zoological Society of London London UK; ^2^ Centre for Biodiversity and Environment Research University College London London UK

**Keywords:** economic growth, gross domestic product, human development, human population density, Sustainable Development Goals, wildlife conservation, wildlife population abundance trends

## Abstract

1. The UN Sustainable Development Goals (SDGs) include economic, social and environmental dimensions of human development and make explicit commitments to all of life on Earth. Evidence of continuing global biodiversity loss has, at the same time, led to a succession of internationally agreed conservation targets.
2. With multiple targets (even within one policy realm, e.g. the CBD Aichi Targets for biodiversity), it is possible for different indicators to respond in the same direction, in opposite directions or to show no particular relationship. When considering the different sectors of the SDGs, there are many possible relationships among indicators that have been widely discussed, but rarely analysed in detail.
3. Here, we present a comparative cross‐national analysis exploring temporally integrated linkages between human development indicators and wildlife conservation trends.
4. The results suggest that in lower income countries there are negative relationships between measures of human population growth and bird and mammal population abundance trends outside protected areas.
5. The results also suggest a positive relationship between economic growth and wildlife population trends in lower income countries. We stress, however, the need for future research to further explore the relationships between economic growth and natural resource‐based imports.
6. Our results highlight a clear potential for compatibility of the conservation and development agendas and support the need for further integration among sustainable development strategies.

A free Plain Language Summary can be found within the Supporting Information of this article.

## Abstract 2

A free Plain Language Summary can be found within the Supporting Information of this article.

## 1 INTRODUCTION

Over the past decades, national leaders have increasingly collaborated internationally to confront humanitarian and environmental challenges, such as eradicating poverty and hunger, slowing the rate of biodiversity loss and preventing further global climate change. These approaches have led to a succession of internationally agreed sustainable development agendas. Despite global adoption, progress towards meeting goals has been slow, and this is often attributed to a lack of integration among policy agendas (CBD, 2010a; Sachs et al., 2009; Walpole et al., 2009). This has led to a growing recognition that neither the conservation nor the development policy agenda can ultimately be realized without progress on the other (Adams et al., 2004; Kaimowitz & Sheil, 2007; Sachs & Reid, 2006).

At present, two concurrent policy agendas are in place: (a) the Convention on Biological Diversity 2020 Aichi Targets for biodiversity (CBD, 2010b) and (b) the UN 2030 Agenda for Sustainable Development, specifying the 17 Sustainable Development Goals (SDGs; United Nations General Assembly, 2015). Both of these global agendas were designed with more coherence in mind than their predecessors, however, the CBD biodiversity targets are in general not likely to be achieved by their deadline in 2020 (Tittensor, Walpole, Hill, & Boyce, 2014), leaving the broader policy objectives of the SDGs, which encompass some aspects of the CBD targets, also at risk.

One explanation for the failing biodiversity agenda is that, even within one policy area (e.g. the Aichi biodiversity targets), there will be instances of both conflict and synergy between achieving different targets, rendering simultaneous achievement of all targets difficult or even impossible (Di Marco et al., 2016; Marques et al., 2014). In broader policy agendas such as the SDGs, synergies and trade‐offs are likely even more pronounced (ICSU, 2017; Machingura & Lally, 2017; Waage et al., 2015). Ignoring these relationships by working to achieve each of the SDG targets in isolation may lead to perverse outcomes, for instance when countries focus primarily on easy to achieve targets while neglecting more systemic problems (Nilsson, Griggs, & Visbeck, 2016). In the biodiversity agenda, better understanding of interactions among targets may improve the efficiency of achieving multiple targets, by avoiding trade‐offs and making use of co‐benefits (Di Marco et al., 2016). In the broader context of wildlife conservation and human development, however, such win–win situations may be rare (Barrett, Travis, & Dasgupta, 2011; Brandon & Wells, 1992; McShane et al., 2011).

The Human Development Index (HDI), a composite indicator of education, life expectancy and per capita income, was shown to be a predictor of wildlife population trends inside protected areas across countries (Barnes et al., 2016), providing evidence for a positive relationship between a broad measure of human development and conservation outcomes. As the authors noted, however, positive wildlife trends inside protected areas may not reflect trends across the wider landscape in a given country, and associations with higher levels of human development may be due to prior extinctions of more human‐sensitive species (i.e. extinction filter effects) (Balmford, 1996). To understand how the interdependencies among wildlife conservation and human development unfold over time, a temporally integrated approach is required that can account for heterogeneous development trajectories between countries (Cumming et al., 2014). Such an approach could provide policy‐relevant information on which aspects of development may be more, or less, compatible with wildlife conservation.

Here, we detail a comparative cross‐national analysis exploring the linkages between temporal trends in human development and wildlife. We use bird and mammal abundance time series for wildlife populations outside protected areas from the Living Planet Database (LPD; WWF, 2018), and a large compendium of national data on social, economic and political indicators of progress towards the SDGs, obtained from the World Bank DataBank (http://databank.worldbank.org/). We employ these data in Bayesian linear regression models to explore how efforts towards achieving the SDGs in low‐ and lower‐middle income countries might covary with bird and mammal population trends. At present, a paucity of spatially and temporally aligned data prohibits comprehensive empirical analysis of compatibility between these societal agendas. Instead, we explore the available data to generate insights that may direct future monitoring and investigation.

Species‐based metrics of trends in wildlife abundance and extinction risk are some of the best‐developed direct measures of biodiversity change (Walpole et al., 2009). Abundance trends provide a more detailed metric of change in status than composite extinction risk measures (Collen et al., 2011), and can provide responsive and accurate indications of biodiversity consequences of anthropogenic pressures. Data in the LPD has been highlighted as providing information on progress towards several CBD Aichi Biodiversity Targets (CBD, 2016), and can provide one informative metric for measuring progress towards biodiversity goals (Mace et al., 2018). We focus on wildlife trends outside protected areas, where competition between conservation and human natural resource use may be most acute. Protected areas usually exclude or restrict natural resource use by people (Adams & Hutton, 2007), and their biodiversity outcomes depend heavily on species‐specific conservation interventions, meta‐population management programmes and overall effectiveness of park management practices (Costelloe et al., 2016; Craigie et al., 2010). Links between wildlife abundance and human development are, thus, likely weaker and more indirect inside than outside protected areas. Moreover, protected areas are not always capable of sustaining large numbers of species (Joppa & Pfaff, 2009; Mcdonald & Boucher, 2011), or catering for all types, especially not for those which are wide‐ranging (Woodroffe & Ginsberg, 1998). For these reasons, we focus instead on better understanding how humans and wildlife can coexist on the broader landscape.

Our analyses present two important analytical advances to cross‐national analyses of this type: (a) they consider a broad spectrum of desirable human progress as proposed in the UN Sustainable Development Agenda in relation to wildlife trends; and (b) they use change over time as their focal metric, avoiding the influence of initial state and instead focusing on progress (or otherwise). In this way, we aim to discriminate the aspects of human development that appear to change independently of wildlife trends, from those where wildlife trends and human development progress are aligned, and those where more scrutiny is required to avoid potential conflicting interests in the future.

## 2 MATERIALS AND METHODS

The analyses performed in this study comprised four distinct parts, which are detailed in the sections below: (a) data preprocessing (Sections 2.1 and 2.2); (b) time‐series modelling (Section 2.3); (c) data homogenization (Section 2.4) and (4) regression modelling (Section 2.5). A schematic of this data processing methodology is also provided in Figure S1.

### 2.1 Wildlife population trend data

As a metric for change in wildlife trends, we use data for bird and mammal population abundance trends from the LPD: a global database of vertebrate population abundance time series collated from scientific literature, online databases and grey literature (Collen et al., 2009; McRae, Deinet, & Freeman, 2017). Time‐series data consisted of population count or density estimates, or proxies for population size, with a minimum length of 5 years and a minimum of two observations per time series, which were comparable in collection method, and had a traceable source; we excluded marine populations to avoid difficulties in assigning these to specific countries. Data were collected and analysed at the global level using standardized methods and approaches, avoiding reliance on national‐level aggregation of wildlife data. Logarithms of population abundance values were calculated for subsequent analyses.

### 2.2 Indicators of progress on the UN SDGs

The UN 2030 Agenda for Sustainable Development comprises 17 SDGs. In March 2016, the UN Statistical Commission agreed to a global indicator framework for the SDGs as a practical starting point for monitoring progress towards their achievement (IAEG‐SDGs, 2016). In this framework, general agreement has been reached on more than 230 SDG indicators. For the purposes of this study, it is essential that SDG indicators match temporally and geographically with the data on wildlife abundance trends. We therefore selected only those with at least 20 years of data and good coverage across countries. Where the officially proposed SDG indicators were unavailable or unsuitable for the purposes of this study, we supplemented them with data from other sources that could serve as a proxy for progress towards the aims of the SDGs, such as the World Governance Indicators for SDG 16 (‘Peace, justice and strong institutions’), and the AidData database (Tierney et al., 2011) for SDG 17 (‘Partnerships for the goals’). For SDG 12 (‘Responsible consumption and production’) and SDG 13 (‘Climate action’), no suitable indicator dataset was found that both spans the temporal and geographical scope of this study and is capable of capturing the likely cross‐national relationships with wildlife abundance. Impacts of the rate of climate change on wildlife abundance have been documented elsewhere (Spooner, Pearson, & Freeman, 2018), and we did not analyse these further here. SDG 15 (‘Life on land’) forms the focus for relationships with all other indicators and SDG 14 (‘Life below water’) was excluded because marine wildlife trends are more difficult to attribute to specific countries than their terrestrial and freshwater counterparts. Metrics of human population density (HPD) and growth (HPG), and the HDI: a composite index of life expectancy, education and economic growth (UNDP, 2015), were added to this set of SDG indicator data to enable comparisons with previous work. In this way, we obtained data on 42 SDG indicators (Table 1). We only included countries that were listed as either ‘low income’ or ‘lower‐middle income’ in the year prior to the start of the study period (i.e. 1995; World Bank, 2016). We limited our analyses to low‐ and lower‐middle income countries to capture wildlife trends contemporaneous with human development, and to avoid measuring signals of historical change including the effects of extinction filters (i.e. capturing mainly trends of more human‐tolerant species) (Balmford, 1996), or overlooking major geographically shifted (cross‐national) impacts (Bunker, 1984; Frey, 1994; York, Rosa, & Dietz, 2003). In more developed countries, links between human development and wildlife abundance may become more indirect and spatially disjunct, as direct dependence on local ecosystems for income and food security becomes weaker (Cumming et al., 2014; Cumming & Cramon‐Taubadel, 2018).

**Table 1:** Sustainable development goal indicators included in this study

SDG	Indicator	Unit	Source	Code
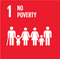	Poverty headcount ratio at $1.90 a day (2011 PPP)	% of population	World Bank, Development Research Group	1.1.
	Poverty headcount ratio at national poverty lines	% of population	World Bank, Global Poverty Working Group	1.2.
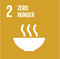	Prevalence of undernourishment	% of population	Food and Agriculture Organization	2.1.
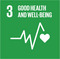	Maternal mortality ratio	Model estimate, per 100k live births	World Health Organization	3.1.
	Mortality rate, under −5	Per 1,000 live births	UN Inter‐agency Group for Child Mortality Est.	3.2.
	Incidence of HIV	% of uninfected pop. ages 15–49	UNAIDS	3.3.
	Life expectancy at birth, total	Years	United Nations Population Division	3.4.
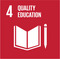	Adult literacy rate, population 15+ years, both sexes	%	UNESCO Institute for Statistics	4.1.
	School life expectancy, primary and secondary, both sexes	Years	UNESCO Institute for Statistics	4.2.
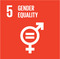	Proportion of seats held by women in national parliaments	%	Inter‐Parliamentary Union (IPU)	5.1.
	Female legislators, senior officials and managers	% of total	International Labour Organization, Key Indicators of the Labour Market	5.2.
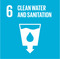	Improved water source	% of population with access	WHO/UNICEF Joint Monitoring Programme for Water Supply and Sanitation	6.1.
	Improved sanitation facilities	% of population with access	WHO/UNICEF Joint Monitoring Programme for Water Supply and Sanitation	6.2.
	Wastewater treatment level weight. by connection to treatment rate	%	Yale Centre for Environmental Law and Policy, Environmental Performance Index	6.3.
	Annual freshwater withdrawals, total	% of internal resources	Food and Agriculture Organization	6.4.
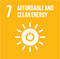	Access to electricity	% of population	World Bank, Sustainable Energy for All	7.1.
	Renewable energy consumption	% of total final energy consumption	OECD/IEA and World Bank	7.2.
	Electricity production from renewable sources, excluding hydroelectric	% of total	OECD/IEA and World Bank	7.3.
	Energy intensity level of primary energy	MJ/$2011 PPP GDP	OECD/IEA and World Bank	7.4.
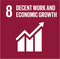	GDP per capita, PPP	Constant 2011 international $	World Bank, International Comparison Program	8.1.
	GDP per person employed	Constant 2011 PPP $	ILO, Key Indicators of the Labour Market	8.2.
	Unemployment, female	nat'l est., % of female labour force	ILO, Key Indicators of the Labour Market	8.3.
	Unemployment, male	nat'l est., % of male labour force	ILO, Key Indicators of the Labour Market	8.4.
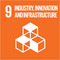	Manufacturing, value added	% of GPP	World Bank national accounts data, and OECD National Accounts	9.1.
	Research and development expenditure	% of GDP	UNESCO Institute for Statistics	9.2.
	Internet users	Per 100 people	International Telecommunication Union, World Telecommunication/ICT Development Report	9.3.
	Mobile cellular subscriptions	Per 100 people	International Telecommunication Union, World Telecommunication/ICT Development Report	9.4.
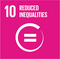	Gini index	World Bank estimate	World Bank, Development Research Group	10.1.
	Income share held by lowest 20%	%	World Bank, Development Research Group	10.2.
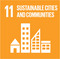	Population living in slums	% of urban population	UN HABITAT	11.1.
	Urban population	% of total	United Nations, World Urbanization Prospects	11.2.
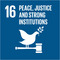	Political Stability and Absence of Violence/Terrorism	Index	Worldwide Governance Indicators	16.1.
	Rule of Law	Index	Worldwide Governance Indicators	16.2.
	Control of Corruption	Index	Worldwide Governance Indicators	16.3.
	Voice and Accountability	Index	Worldwide Governance Indicators	16.4.
	Government Effectiveness	Index	Worldwide Governance Indicators	16.5.
	Regulatory Quality	Index	Worldwide Governance Indicators	16.6.
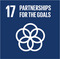	International Aid for Biodiversity purposes	USD	AidData	17.1.
	International Aid for non‐biodiversity purposes	USD	AidData	17.2.
NA	Human population density	People/km^2^ land	World Bank World Development Indicators	HPD
	Human population growth	Annual %	World Bank World Development Indicators	HPG
	Human Development Index	Index	UNDP	HDI

For SDG indicators that were estimates of population size or had monetary units, variables were log‐transformed, to overcome distribution skewedness. Similar to wildlife abundance time series, SDG indicators for a particular country were included only if they had at least two observations per time series. In case SDG indicators contained negative values, the indicator was centred to an entirely positive distribution by subtracting the negative, minimum value of the indicator series across countries, from the whole indicator series.

### 2.3 Time‐series models

Following Collen et al. (2009), both wildlife abundance and SDG indicator trends were modelled using two different methods. For times series with *n* < 6, linear models of indicator value over time were derived in the r ‘stats’ package (R Core Team, 2018). For *n* ≥ 6, a Generalized Additive Model (GAM) was derived in the r ‘mgcv’ package (Wood, 2006) to better account for nonlinearity in longer time series (Collen et al., 2009). In the GAM procedures, the model smoothing parameter was set to half the length of the time series. For linear models, missing values were imputed with linear interpolation using the r ‘zoo’ package (Zeileis & Grothendieck, 2005); in the GAM procedure values were predicted from model parameters. For each complete time series, annual change values were calculated as the change in (predicted) values between subsequent years. Annual change values were retained for the study period (1996–2015) if: (a) they came from models with good fit (*R* ^2^ > 0.5) and (b) total time‐series length within the study period was 5 years or longer. For each of these remaining annual change time series, the mean was taken over the study period as the final wildlife abundance or SDG indicator trend. Each wildlife abundance trend was then merged with the stack of available SDG indicator trends in that country.

### 2.4 Homogenization of country subsets across SDG indicators

Data gaps affecting SDG indicators were strongly misaligned among countries in which wildlife abundance trends were recorded. Total country samples and sample sizes were therefore not consistent across SDG indicators. For birds, the effective sample size ranged from 27 to 164 populations across 11–35 countries; whereas for mammals, this was 51–165 populations across 11–28 countries. Because of this variation, we limited our analyses to a smaller, homogenized sample of countries and indicators for which we could align the countries and wildlife populations across SDG indicators. In this process, we strived to balance the exclusion of data‐poor countries with the exclusion of data‐poor indicators, to arrive at a complete set of data across a reasonable number of indicators, in a reasonable number of countries. Countries were thus homogenized across indicators by first removing any very data‐poor countries (*N*_indicators_ < 21), retaining those that had data for at least half the total number of SDG indicators. Remaining countries after this step could still have (misaligned) data gaps. Subsequently, we excluded any very data‐poor SDG indicators, with which we could associate fewer than 160 wildlife populations. Each of the remaining final indicators was then associated with at least 160 wildlife populations, but these were not necessarily the same ones across indicators. Therefore, as a final step, we excluded any remaining countries that did not have a complete set of data on the full final set of SDG indicators, after these first two steps. These procedures were designed to maximize the number of countries shared across the largest number of SDG indicators, to enable investigation of the relationships between SDG indicators and wildlife trends that hold most‐widely across a consistent set of countries and wildlife populations.

### 2.5 Regression models

SDG indicator trends were normalized and in some cases inverted (multiplied by −1), so that, for all SDG indicators greater SDG indicator values indicated greater progress towards the SDG regardless of the specific phrasing of the indicator (e.g. the proportion of the urban population living in slums was inverted to obtain a measure of the proportion of the population living in adequate, safe and affordable housing). We first assessed relationships among SDG indicators by calculating Pearson product‐moment correlations with the r ‘Hmisc’ package (Harrell, 2014). Because many SDG indicators are highly correlated (Figure S2), and because we were interested in associations between wildlife abundance trends and individual development indicators, we modelled correlations between bird and mammal abundance trends and changes in SDG indicators in univariate generalized linear mixed models (GLMMs), rather than deriving more complex models that would explain the greatest amount of variance in the wildlife trends. GLMMs were thus used as vehicles for assessing correlations while accounting for random effects. Models were derived in a Bayesian framework using the R implementation of the Integrated Nested Laplace Approximation (INLA) (Rue, Martino, & Chopin, 2009). A Bayesian approach can provide credible information even under small sample sizes and has been shown to deal better with the presence of dependent outliers (Fong, Wakefield, Rosa, & Frahm, 2012). The r ‘INLA’ package (Martins, Simpson, Lindgren, & Rue, 2013) provides a fast and accurate implementation of Bayesian analysis for estimating regression coefficients in models with complex and independent random error structures, as an alternative to simulation‐based Monte Carlo Markov Chain procedures (Fong, Rue, & Wakefield, 2010). Here, we exploited these benefits to investigate the evidence for relationships between SDG indicators and wildlife abundance trends, while accounting for independent random errors at the species level (thus accounting for differences in trends of populations of the same species), at the taxonomic order level (to account for potential differential responses of different taxonomic groups to anthropogenic pressures), and for different geographical regions (to control for geographical non‐independence of countries). Additional linear effects were included for (log‐transformed) country land area and (log‐transformed) HPD (year 1996; to account for country‐level scale and demographic effects). We used default uninformative Bayesian prior parameter estimates (*μ* = 0, *σ^2^* = 1,000) for all SDG indicators.

## 3 RESULTS

**Figure 1:**
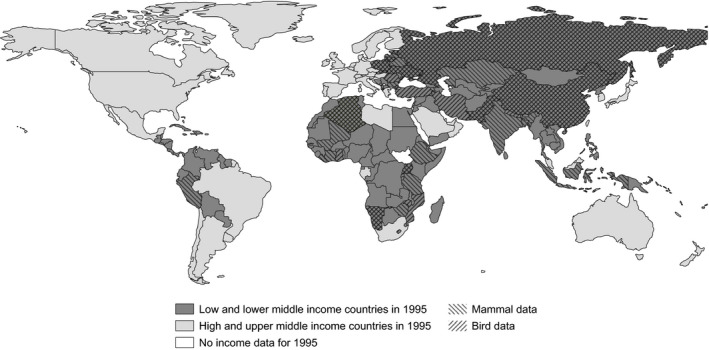
Countries by UN income class in 1995. Countries with bird (*n* = 19) and mammal (*n* = 26) population abundance trends used in this study shown in hash patterns. Country samples provided as lists in Table S1

The LPD contains a substantial number (18,264) of vertebrate population time series, but after applying all necessary constraints to ensure quality and homogenization (see Figure S1 for a process summary), we obtained trends for 147 bird populations and 151 mammal populations, in 19 and 26 countries, with 22 and 20 SDG indicators respectively. Bird data comprised 11 taxonomic orders from countries in Europe, Asia and Africa; mammal data comprised 11 taxonomic orders from Latin America and the Caribbean, Europe, Asia and Africa (Figure 1, and Figure S3 and Table S1).

**Figure 2:**
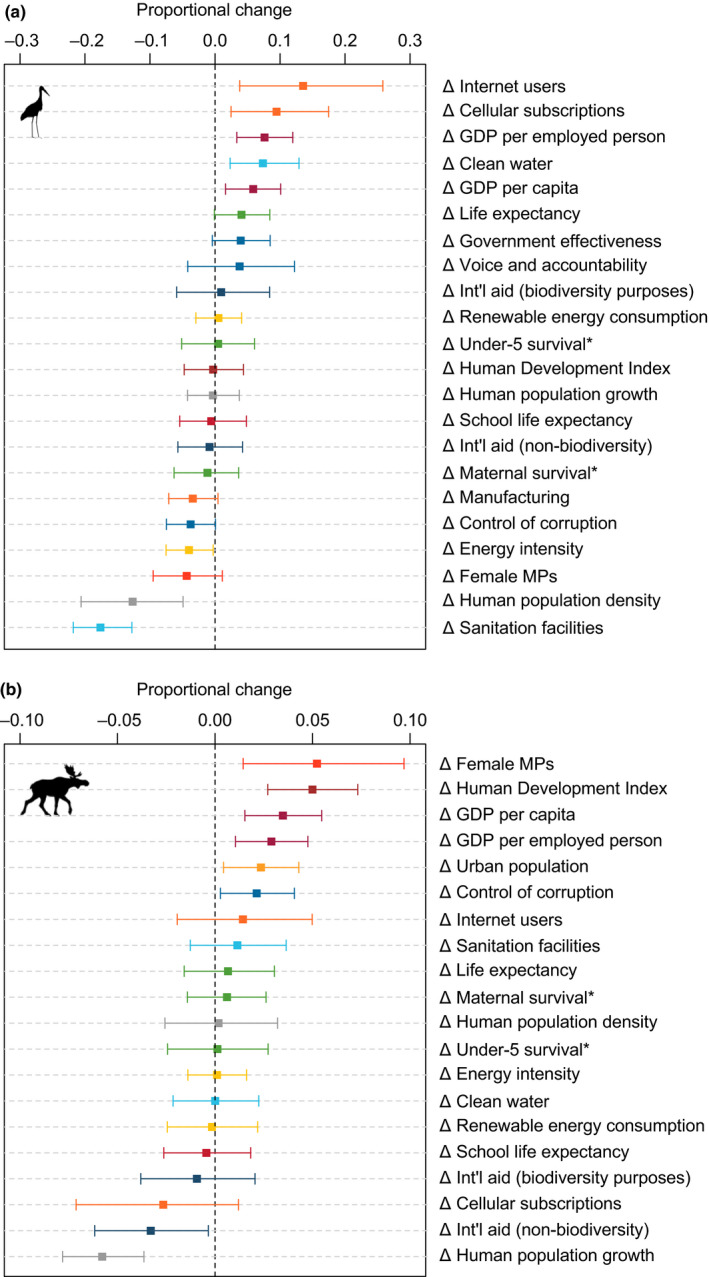
Proportional change in (a), bird abundance trends (*n*_countries_ = 19, *n*_populations_ = 147) and (b), mammal abundance trends (*n*_countries_ = 26, *n*_populations_ = 151) outside protected areas corresponding to a one *SD* increase in the change of SDG indicator levels in developing countries between 1996 and 2015. Indicators appended with an asterisk were inverted (i.e. multiplied with −1), so that for each indicator positive change signifies desirable progress. Error bars represent 95% credible intervals from posterior distributions of regression coefficients. Indicators coloured by SDG (see Table 1)

For bird abundance trends, we found support for relationships with eight out of 22 SDG indicators (Figure 2a), where 95% credible intervals of posterior distributions of regression coefficients did not overlap a zero relationship. Positive relationships were found with indicators of economic and technological development: change in (log‐transformed) gross domestic product (GDP) per capita and per employed person, change in Internet and mobile cellular network usage as a proportion of the population, and with change in the proportion of the population with access to clean water. Negative relationships were found between bird abundance trends and change in the proportion of the population with access to safely managed sanitation facilities, for change in (log‐transformed) HPD, and very marginally for change in the energy intensity of the economy (Figure 2a).

For mammal abundance trends, results indicated noteworthy relationships for eight out of 20 SDG indicators (Figure 2b). Mammal abundance trends were positively correlated with a change in the HDI, change in (log‐transformed) GDP per capita and per employed person, change in the proportion of the population living in cities, change in the level of corruption control, and with a change in the proportion of seats in national parliaments held by women. Negative relationships with mammal abundance trends existed very marginally with the change in cumulative international aid for non‐biodiversity purposes and with change in the rate of HPG (Figure 2b).

In total, this study provides evidence for relationships between change in wildlife abundance trends and 14 out of 23 tested Sustainable Development Goal indicators. For bird abundance, our results showed the strongest positive relationships with indicators of SDG 8 (‘decent work and economic growth’) and SDG 9 (‘industry, innovation and infrastructure’). The strongest negative relationships were found for indicators of SDG 6 (‘clean water and sanitation’) and change in HPD.

For mammals, results also provided the strongest support for positive relationships with SDG 8 (‘decent work and economic growth’), but also showed evidence for relationships with progress on SDG 9 (‘gender equality’). In addition, we found a positive relationship between change in mammal abundance trends and change in HDI. The strongest negative relationships with mammal population abundance trends were found for change in HPG rate.

## 4 DISCUSSION

In this exploratory, cross‐national analysis of linkages between human development and wildlife abundance, we found consistent positive relationships between indicators of economic growth (GDP per capita and GDP per employed person) and wildlife abundance trends (Figure 2a,b), across birds and mammals. Negative relationships were found between metrics of HPG and wildlife population trends. For birds, our results show negative relationships with change in HPD (Figure 2a), whereas for mammals, negative relationships were found with change in the HPG rate (Figure 2b). These results are consistent with the neo‐Malthusian viewpoint that HPG may compete with biodiversity for space and resources, through increasing demands for agricultural land‐use and infrastructure development (Crist, Mora, & Engelman, 2017; Ehrlich & Holdren, 1971; Holdren & Ehrlich, 1974). Similar relationships have been found between HPD and the ecological footprint (Dietz, Rosa, & York, 2007; York et al., 2003), and extinction risk in (large) mammal (Brashares, Arcese, & Sam, 2001; Cardillo et al., 2005; Hoffmann, 2004; McKee, Chambers, & Guseman, 2013), fish (Clausen & York, 2008), bird (Hoffmann, 2004; McKee et al., 2013) and carnivore species (Cardillo et al., 2004).

We also found a positive association between mammal abundance trends and the changes in HDI (a composite indicator of life expectancy, education and economic growth; Figure 2b). This suggests that the positive associations of HDI differences over space identified previously (Barnes et al., 2016), are not simply an artefact of using a space‐for‐time substitution. This partially dispels the suggestion that these associations are due to prior extinctions of more human‐sensitive species (i.e. extinction filter effects).

Our results show consistent positive relationships between two indicators of economic growth (i.e. GDP per capita and GDP per employed person) and both bird and mammal abundance trends (Figure 2a,b), and a positive association between mammal abundance trends and the HDI. In combination, these results may align with expectations from Ecological Modernization Theory (Mol, 1996; Mol & Spaargaren, 2000) in environmental sociology, which suggests that as countries develop economically, industry becomes more tightly regulated, individuals place higher priority on environmental quality, and the advancement of modern institutions ultimately reduces (or exports) the environmental impacts associated with capitalism (York et al., 2003). Our results suggest that increases in national economic affluence are, in fact, associated with positive changes in wildlife abundance at the national scale (Figure 2), thus potentially supporting this statement.

An important branch in Ecological Modernization is the work around the Environmental Kuznets Curve (EKC) (Grossman & Krueger, 1994; Stern, Common, & Barbier, 1996): a postulated inverted‐U shaped relationship between environmental degradation and economic development. Although we did not test for the existence of an EKC in our data, by including only countries at a common baseline level of human development (i.e. countries listed as low‐ and lower‐middle income at the start of the study period), we believe we were able to control for potentially changing relationships between social indicators and wildlife abundance trends at different levels of development (see Section 2.2). Limiting our analyses to these countries not only served as a control against compounded results due to the possible existence of an EKC, but also helps reduce the effects of historic extinction filters or geographically shifted impacts in more developed countries that largely outsource their natural resource extraction (Ehrlich & Holdren, 1971; York et al., 2003). We acknowledge, however, that this is not a perfect solution, as, even in developing countries, international trade could contribute to around 30% of the number of threatened species (Lenzen et al., 2012), and is estimated to displace 1.8 billion global hectares of land use (24% of the global land footprint) (Weinzettel, Hertwich, Peters, Steen‐Olsen, & Galli, 2013). Considering the embedding of societies and economies within the biosphere (Folke, Biggs, Norström, Reyers, & Rockström, 2016; Waage et al., 2015), this analysis suggests that there remain important macroecological and macroeconomical relationships to be explored. In particular, our results suggest that in lower‐income countries, there may certainly be scope for human development whilst sustaining free‐roaming wildlife.

We note, that within the current framework, we were unable to account for important relationships between economic growth and increasing resource‐based imports, which could potentially be a substitute for impacts on wildlife trends nationally. As countries grow economically, quantifying how their cross‐national environmental impacts subsequently change will be critical.

The objective of ensuring declining environmental impact with increasing economic growth has been embraced in policy terms as resource and impact decoupling (UNEP, 2011). These concepts postulate the desired dissociation of economic and population growth from environmental impact. The results reported here support this model insofar that higher economic growth was associated with more positive bird and mammal population abundance trends, but support for any decoupling of population growth and environmental impact was lacking in our findings. Cumming and Cramon‐Taubadel (2018) show how feedback loops between societies and natural resources can give rise to such results, suggesting that (rural) HPG leads to further reliance on ecosystems for income and food security. Although our results show consistent positive relationships between wildlife population abundance trends and growth in per capita income, Marques et al. (2019) recently showed how increases in per capita income could correspond to declines in bird species richness across Asia, Africa and Central and South America. The findings reported here are cumulatively supported by observed data on 298 wildlife populations across 33 countries, whereas Marques et al. infer species richness from species‐area relationships. It is important to recognize that biodiversity has many dimensions (Reyers, Stafford‐Smith, Erb, Scholes, & Selomane, 2017), and alternative measures may tell a different part of the full story of biodiversity change in response to anthropogenic change. In our models we do not account for habitat loss directly, which may give rise to these different results.

Similarly, our results indicate negative relationships between measures of HPG and wildlife trends across a cumulative 298 wildlife populations in 33 countries. These results are consistent across bird and mammal populations. This, coupled with the consistent results for economic growth, suggests a consistency of ecological responses in these data, but as more data become available, more careful analyses may be used to further test these hypotheses. Our analyses reveal some differences in relevant metrics between bird and mammal populations, such as the HPD and HPG metrics. Seemingly inconsistent results between taxa are not entirely uncommon in the literature (e.g. Naidoo & Adamowicz, 2001). In the present analysis, country samples were not identical between the bird and mammal datasets (Figure 1, Table S1), and thus, direct comparisons between these results may be inappropriate.

We also found a slight positive relationship between the change in the percentage of the population living in urban environments and mammal population abundance trends, potentially indicating national‐level environmental benefits of urbanization, and competing demands for land‐use in more natural resource‐based, rural economies. Others have argued that in addition to changes in overall HPD and levels of urbanization, household size and numbers (Liu, Daily, Ehrlich, & Luck, 2003), as well as demographic transitions (York et al., 2003) are important predictors of environmental impact.

Interestingly, no relationships were found between the change in cumulative international aid for biodiversity purposes, and either bird or mammal population trends. With a vast array of plausible conservation funding strategies, a lack of a consistent relationship may not be entirely surprising. One strategy could be that relatively more conservation aid is spent in places with rapidly declining wildlife abundance trends to help reverse those trends (e.g. species‐specific aid for white rhinoceros *Ceratotherium simum*); or alternatively, relatively more conservation aid may be spent in places showing positive results (recovering populations) after a successful pilot project (evidence‐based approaches). If these and other strategies exist side‐by‐side, a correlation across countries may not find conclusive evidence for any one strategy.

While it might seem counter‐intuitive that improvements to sanitation and wastewater treatment reflect poorly on birds (Figure 2a), a large proportion of bird populations in the dataset are water‐dependent species (Figure S3), and negative effects of the construction of sanitation and wastewater treatment plants are apparent on some freshwater bird species, particularly when development results in concentrated discharges of wastewater into natural waterbodies (Alves, Sutherland, & Gill, 2011; Carey & Migliaccio, 2009; Mallin, Williams, Esham, & Lowe, 2000). More detailed analyses on these and other data are required to confirm whether this is indeed the mechanism underlying these results.

A major limitation in our analyses was the availability of appropriate long‐term data. The framework for SDG indicators put forward by the Inter‐Agency and Expert Group on Sustainable Development Indicators (2016) may be useful as a conceptual gold standard; in practice, however, proposing 230 indicators may lead to a dilution of monitoring efforts and to nations pursuing different metrics, severely restricting any macro‐level analysis. In our analyses, we found that SDG indicator data are currently not available for all countries in which wildlife trends are available. We limited our analyses to a consistent set of countries and wildlife populations across indicators, but this sample may have inherited bias in the availability of national datasets. In addition, some of the SDG indicators may also seem quite far removed from what really matters in societies. The SDG indicators are proposed based on the appropriateness of their content, clarity of methodology and ease and consistency of data collection, but ultimately, agreement on their official inclusion is reached through a political process. Given these methodological, political and availability constraints, the final indicator set employed here provides a far from comprehensive picture of potential connections between human development and wildlife conservation. Further efforts should be mounted to improve data coverage, representativeness and integration of both SDG and biodiversity indicators, enabling further insight into their interconnections in the future.

## 5 CONCLUSIONS

This study provides the first empirical evidence for relationships between wildlife trends outside protected areas, and progress in achieving the UN SDGs in lower‐income countries. Using well‐established datasets for 298 populations in 33 countries, the results provide new evidence for relationships between wildlife trends and economic and demographic change. They identify several associations that merit focussed investigation and formal testing in future work. The combination of a positive association of wildlife abundance trends with economic growth, and a negative association with HPG, suggests that there are complex interactions between the conservation and development agendas at the landscape‐level. Focussing on wildlife outside protected areas, we show a clear potential for compatibility of free‐roaming wildlife with sustainable development, but considering more explicitly indicators that reflect potentially competing land‐use practices will be important in future work. Clarifying the local mechanisms that generate taxonomic and regional differences would also be beneficial. In addition, we recommend future research to further explore the potential cross‐national environmental impacts associated with economic growth. Further research effort should also identify the characteristics of countries where relationships play out favourably, such as countries where both economic affluence and wildlife population abundances increased, as opposed to countries where affluence and wildlife population abundances declined, since both these scenarios elicit the same statistical relationship. Exploration of specific conservation and development strategies responsible for co‐occurring desirable social and environmental outcomes will be beneficial for securing these in more places around the world.

There is ample reason to believe local conditions mitigate and amplify these interactions, and this effort must thus be viewed as providing preliminary conceptual validation, rather than delivering definitive empirical conclusions. Despite this, our results demonstrate a clear potential for compatibility of the conservation and development agendas and underline the need for further integration of sustainable development strategies. Importantly, our results highlight the complex interactions between economic development, population growth and biodiversity trends.

## CONFLICT OF INTEREST

The authors declare no conflict of interest.

## AUTHORS' CONTRIBUTIONS

J.M.A. and B.C. conceived the study; J.M.A., B.C. and R.F. designed the methodology; R.F. provided data on wildlife abundance trends; J.M.A. acquired additional datasets on SDG indicators, performed analyses and wrote the initial draft. All authors contributed critically to data interpretation and manuscript revision and gave final approval for publication.

## Data Availability

The data that support the findings in this study are publicly available. The SDG indicator data are available from the World Bank DataBank (https://databank.worldbank.org/data/source/sustainable-development-goals-(sdgs)). Wildlife abundance trends used in this study are available from the Living Planet Database (http://www.livingplanetindex.org/data_portal).
