# Supplementary material for: Compatibility between agendas for improving human development and wildlife conservation outside protected areas: Insights from 20 years of data
Source: People Nat (Hoboken). 2019 Aug 1;1(3):305–16. doi: 10.1002/pan3.10041 (PMC8641387; doi:10.1002/pan3.10041)
Supplement: Supplementary file 1 [file PAN3-1-305-s002.pdf]

PEOPLE AND NATURE

Wildlife conservation can be compatible with human development, but increasing interactions with humans may pose problems

Judith M. Ament, Ben Collen, Chris Carbone, Georgina M. Mace, and Robin Freeman

Countries around the world have agreed to work towards improving conditions for people and the environment by the year 2030. These plans are laid out in 17 Sustainable Development Goals, which contain broad societal ambitions such as reducing poverty, improving healthcare, and tackling inequalities. At the same time, countries have also united against biodiversity loss, by committing to the Aichi Biodiversity Targets. Taken together, these ideals for biodiversity and human development could generate conflicting interests for land and resources. With so many aspirations in place, it may not be possible to achieve all targets simultaneously. In this study, we analysed 20 years of data to understand whether progress towards development goals in lower-income countries tends to align with improvements in wildlife conservation outside protected areas. We use a large dataset of country-level information on the Sustainable Development Goals collated by the WorldBank, in conjunction with a large dataset on wildlife population abundance timeseries, to assess changes in human development and biodiversity over a 20-year period. Our results suggest that in lower-income countries, there is a positive relationship between economic growth and bird and mammal population abundance trends outside protected areas, while measures of human population growth show negative relationships with wildlife population trends. We find that data for human development and biodiversity are not always well-aligned in space and time. Greater effort is needed to ensure wider coverage, representativeness and integration of biodiversity and development datasets. Our results demonstrate the clear potential for compatibility of the conservation and development agendas and underline the need for further integration of sustainable development strategies. Importantly, our results highlight the complex interactions between economic development, population growth and biodiversity trends.

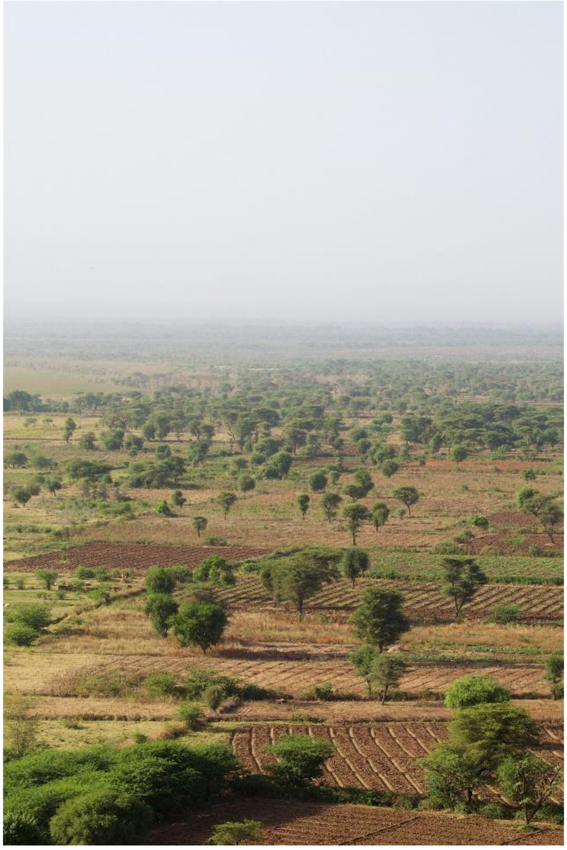

Mixed-use landscape in Kenya, illustrating the potential conflicts between human expansion and free-roaming wildlife. Photo credit: Robin Freeman, 2019.
