# Supplementary material for: Compatibility between agendas for improving human development and wildlife conservation outside protected areas: Insights from 20 years of data
Source: People Nat (Hoboken). 2019 Aug 1;1(3):305–16. doi: 10.1002/pan3.10041 (PMC8641387; doi:10.1002/pan3.10041)
Supplement: Supplementary file 2 [file PAN3-1-305-s001.pdf]

Supporting Information

Compatibility between agendas for improving human development and wildlife conservation outside protected areas: insights from 20 years of data

Judith M. Ament, Ben Collen, Chris Carbone, Georgina M. Mace, and Robin Freeman

Correspondence: judith.ament@ioz.ac.uk; robin.freeman@ioz.ac.uk

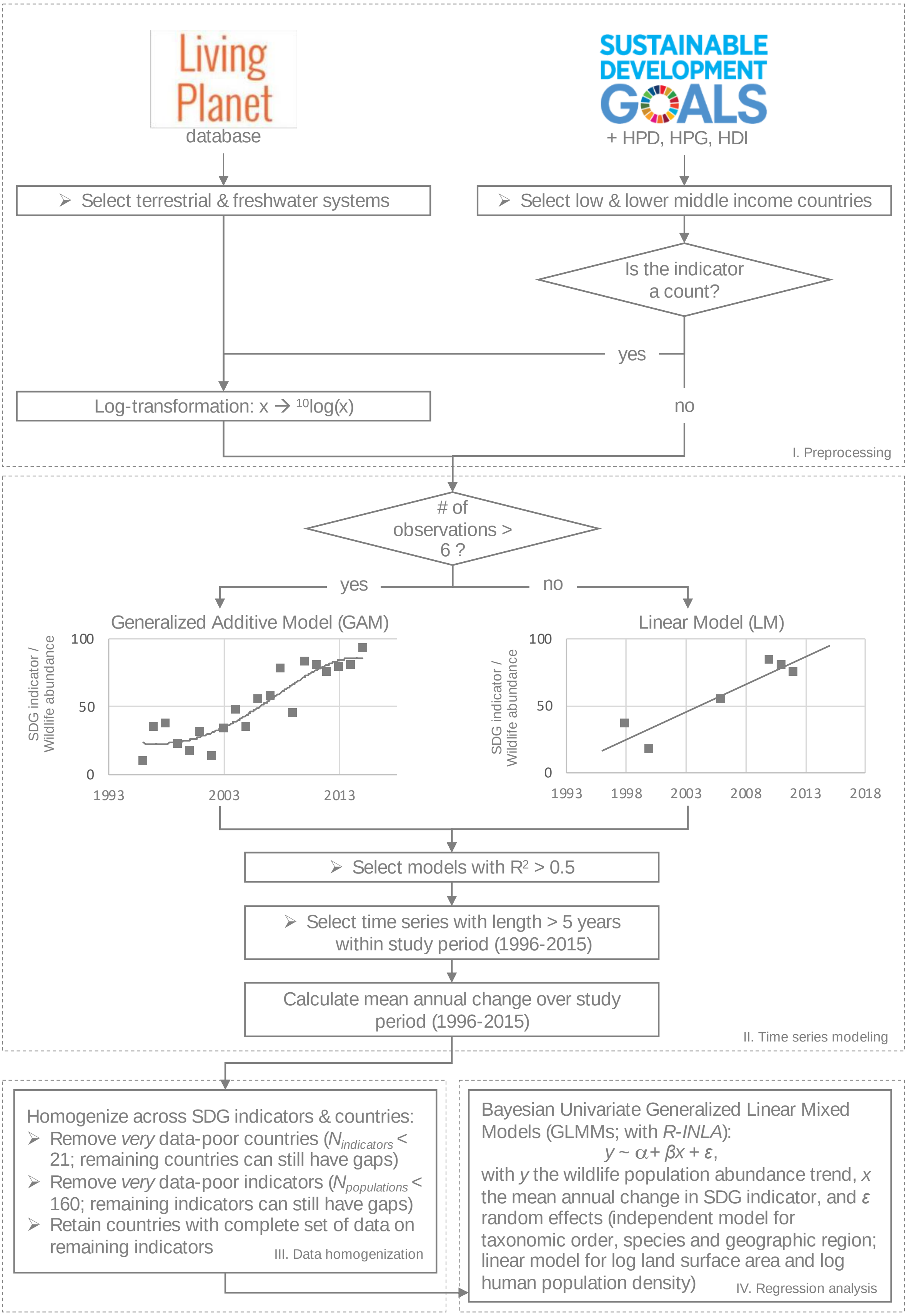

Fig. S1. Schematic representation of the data processing methodology employed in the study

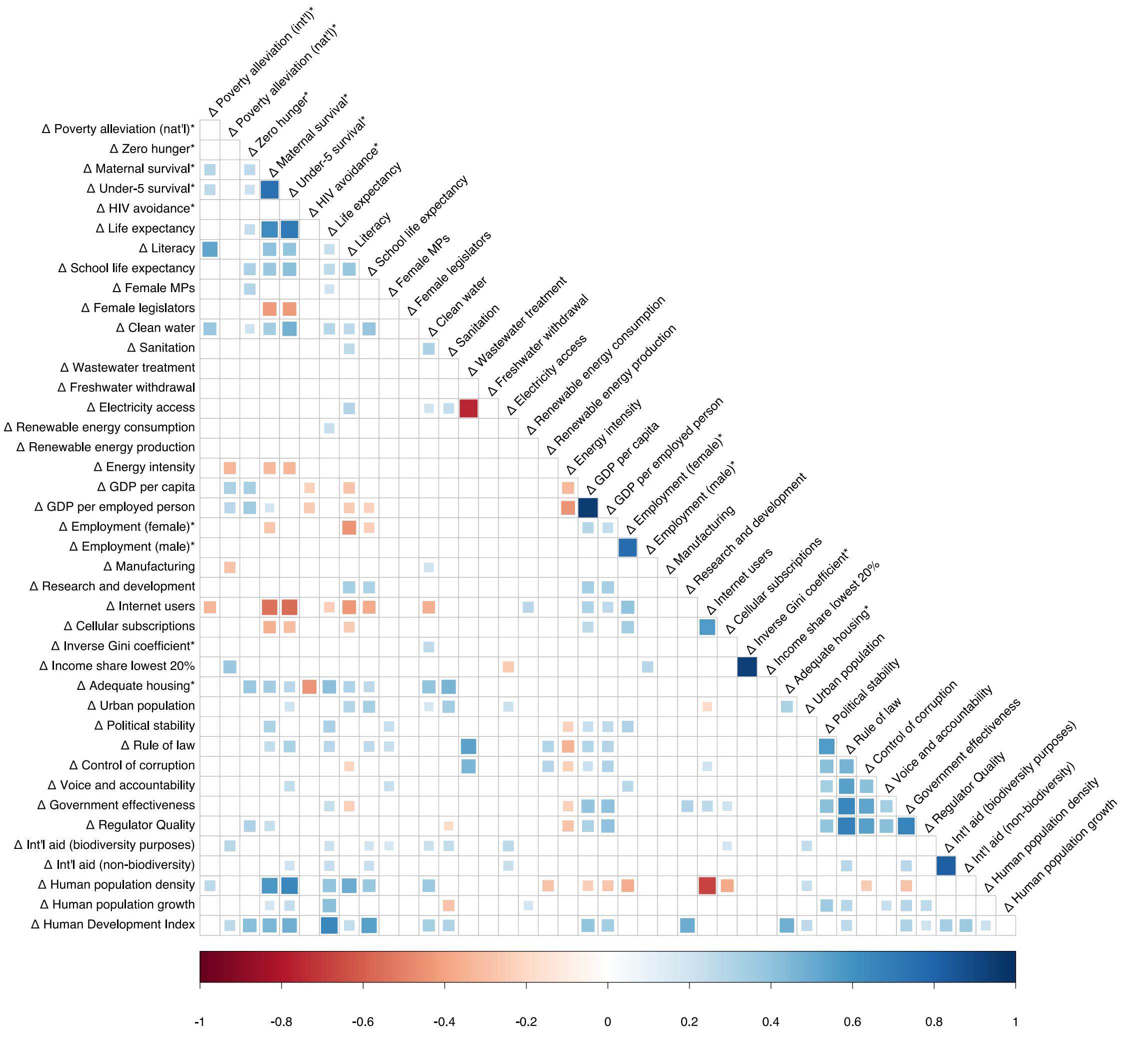

Fig. S2. Pearson correlations between changes in Sustainable Development Goal indicators between 1996 and 2015. Correlation strength indicated by size and shade of cells, showing positive (blue) and negative (red) correlations with $p < 0.05$. For indicator names appended with an asterisk the original indicator values were inverted (i.e., multiplied with -1), so that positive change signifies desirable progress towards the SDG.

a

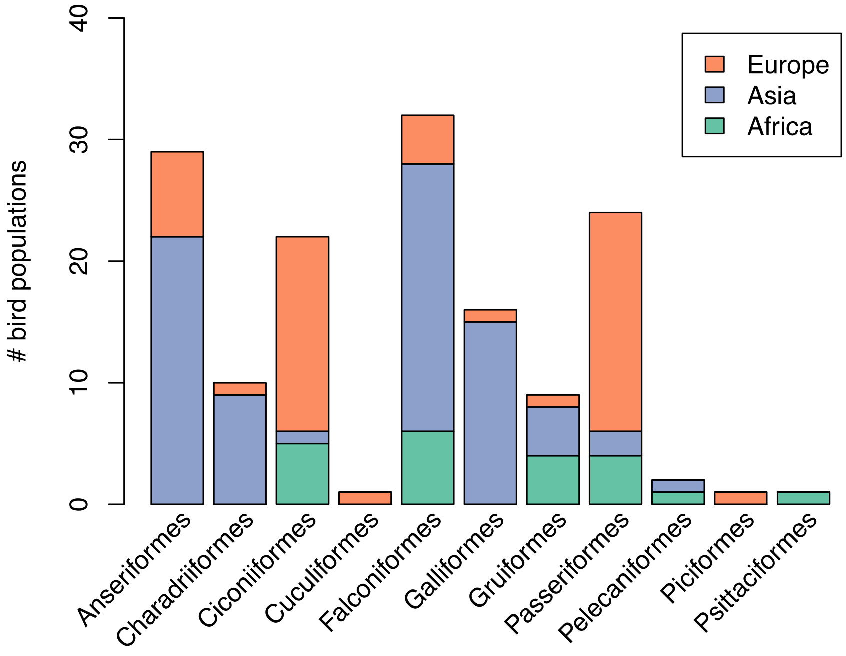

b

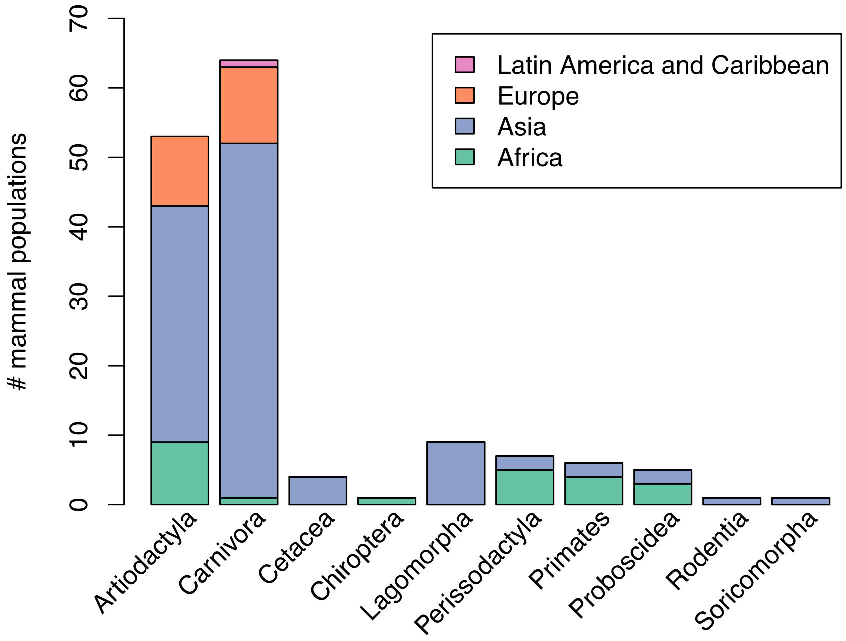

Fig. S3. Numbers of populations included in the analysis. Populations available by taxonomic order and geographic range for **a**, birds and **b**, mammals, from Latin America and the Caribbean (pink), Europe (orange), Asia (dark blue), and Africa (green).

Table S1. Countries included in the analyses.

Countries	In bird	In mammal
Albania	✓	✓
Algeria	✓	✓
Bangladesh		✓
Belarus		✓
Bulgaria		✓
China	✓	✓
Cote d'Ivoire		✓
Estonia	✓	✓
Ethiopia		✓
Ghana	✓	
India		✓
Iran, Islamic Rep.	✓	
Indonesia		✓
Kazakhstan		✓
Latvia		✓
Lesotho	✓	
Liberia		✓
Lithuania	✓	✓
Mali		✓
Mozambique	✓	
Namibia	✓	✓
Nepal	✓	
Pakistan	✓	✓
Peru		✓
Poland	✓	✓
Romania	✓	
Russian Federation	✓	✓
Slovak Republic	✓	✓
Tanzania		✓
Turkey	✓	
Uganda	✓	✓
Ukraine	✓	✓
Zimbabwe		✓
